# Efficacy of Tuina Therapy Based on Hip-Knee-Ankle Synergy in the Lower Extremity Kinematic Chain in Patients With Knee Osteoarthritis: Protocol for a Randomized Controlled Trial

**DOI:** 10.2196/75547

**Published:** 2025-07-07

**Authors:** Zonglin Wen, Ruoyun Lyu, Shuaipan Zhang, Yifeng Yu, Jianwei Wang, Lingjun Kong, Min Fang

**Affiliations:** 1 Department of Tuina Shuguang Hospital Shanghai China; 2 Department of Traditional Chinese Medicine Tenth People’s Hospital of Tongji University Shanghai China

**Keywords:** KOA, knee osteoarthritis, Tuina, hip-knee-ankle synergies, kinematic chain, clinical trial

## Abstract

**Background:**

Knee osteoarthritis (KOA) is a progressive degeneration of the knee joint that has the potential to impair the function of the lower extremity. Tuina, as an important component of traditional Chinese medicine (TCM), is a common treatment for KOA. However, the prevailing practice often limits the therapeutic focus to the knee joint, overlooking potential coexisting hip and ankle injuries. Thus, the therapeutic efficacy may not be optimal in these instances.

**Objective:**

This study aims to evaluate the clinical efficacy of Tuina therapy based on hip-knee-ankle synergy combined with conventional treatment in patients with KOA. It also seeks to explore the potential effects on the lower extremity kinematic chain and provide clinical practitioners with a potentially valuable therapeutic approach.

**Methods:**

This single-blind randomized controlled trial (RCT) will involve a total of 96 participants with KOA from 1 study center (including 2 branches). Participants will be randomly divided into a conventional treatment (control) group and a Tuina+conventional treatment (intervention) group in a 1:1 ratio. Participants in the control group will receive regular health education, self-administered acupressure, and functional exercise. This routine will be completed in 4 weeks, with a total of 5 sessions planned each week. Participants in the intervention group will receive 8 sessions of Tuina therapy based on hip-knee-ankle synergy (twice a week for 4 weeks), combined with conventional treatment. The primary outcome of this study will be improvement in WOMAC (Western Ontario and McMaster Universities Osteoarthritis Index) scores from baseline to 16 weeks. Among the secondary measures, the Key Symptoms and Signs/TCM Syndrome Classification Quantitative Evaluation of KOA and the 11-item Tampa Scale of Kinesiophobia (TSK-11) score will be evaluated from baseline to 16 weeks. The Functional Movement Screen (FMS), the Timed Up and Go (TUG) test, and the Weight Bearing Lunge Test (WBLT) scores will only be evaluated at baseline and 4 weeks.

**Results:**

The trial commenced in September 2024 and is expected to conclude in September 2025. As of April 2025, key preliminary steps have been successfully completed. Specifically, the ethical review and clinical trial registration have been finalized. Participant recruitment is proceeding well, with 69 individuals having been enrolled to date. Data collection is currently ongoing, and formal data analysis has not yet been initiated.

**Conclusions:**

This RCT aims to contribute to the growing evidence base supporting the necessity of kinetic chain–based integrative diagnosis and treatment, while also evaluating the feasibility of the study protocol and exploring the benefits of manipulative therapy.

**Trial Registration:**

International Traditional Medicine Clinical Trial Registry ITMCTR2024000293; https://tinyurl.com/4tsszfdu

**International Registered Report Identifier (IRRID):**

DERR1-10.2196/75547

## Introduction

Knee osteoarthritis (KOA) [1,2] is a common musculoskeletal problem worldwide, characterized by pain, stiffness, swelling, and limited joint function. According to radiographic confirmation [3], KOA is estimated to affect about 3.8% of the world’s population. The incidence of this condition increases with age, with prevalence exceeding 10% in individuals over the age of 60 years, especially women. Several lifestyle factors [4,5] are known to heighten the risk of developing KOA. These include increased body mass, specific occupational roles that involve repetitive knee stress, and a history of traumatic knee injuries.

Although many guidelines [6-8] recommend physical exercise to improve pain and function in patients with KOA, pain and a fear of exercise injury are also major barriers to adherence to exercise. Only a few patients can follow the guidelines. As an additional option, Tuina therapy has become one of the preferred treatment methods for many patients. However, it is crucial for many clinicians to recognize the integral role of hip and ankle synergistic joints in knee joint injuries. These synergistic injuries may occur simultaneously or sequentially with knee joint injury, and their diagnosis and treatment should not be overlooked [9].

Traditional Chinese medicine (TCM), with its long-standing history and unique theoretical system, has garnered increasing attention for its role in managing KOA. Recent advances in translational Chinese medicine have further illuminated the potential of TCM-based approaches in addressing KOA. A study has shown that acupotomy can effectively upregulate the expressions of adenosine monophosphate–activated protein kinase (AMPK), Unc-51-like autophagy-activating kinase 1 (ULK1), and Beclin1; downregulate the expression of mechanistic target of rapamycin (mTOR); promote autophagy; and alleviate joint degeneration [10]. This highlights the complex mechanisms through which TCM influences KOA pathogenesis and progression. A network pharmacology study demonstrated that paeoniflorin exerts potent anti-inflammatory effects on synovial inflammation in osteoarthritis (OA) [11]. These findings bridge TCM and modern pharmacological approaches, offering novel insights into the development of effective TCM-based therapies for KOA. Additionally, research on Tai Chi outlines its benefits in alleviating physical impairments in patients with KOA, including improvement in muscle strength function, proprioception, and sleep function and reduction in joint mechanical stress, pain, and inflammation [12]. These studies further corroborate the efficacy and feasibility of TCM interventions in KOA management.

TCM [13] is highly influenced by the concept of viewing things in classical Chinese philosophy. The concept of holism permeates all areas of TCM, including physiology, pathology, diagnosis, differentiation, and treatment of the syndrome. TCM believes that the human body is an organic whole, including the hips, knees, and ankles. Indeed, the concept of holism is an essential component of TCM and underlies the practice of Tuina therapy. Therefore, the principle of integrity should be implemented in the treatment of KOA, and comprehensive judgment and holistic treatment should be emphasized [14].

To explore the scientific model of the interaction between bones, muscles, and joints and their functional transformation rules during human movement, modern medicine has proposed the concept of the kinematic chain. The synergistic effect of the lower limb kinematic chain, consisting of hip, knee, and ankle joints, is of paramount importance in the process of human movement. Any abnormality in the lower limb’s kinematic chain can lead to joint pain, muscle weakness, poor posture, and other complications, which can negatively affect an individual’s exercise performance and overall quality of life [15,16]. Therefore, in the treatment of KOA, we should not only focus on the local knee but also consider the corresponding changes in the hip and ankle and implement a targeted therapeutic approach.

Therefore, we hypothesize that Tuina has the potential to ameliorate clinical symptoms of KOA by correcting abnormalities in the lower limb kinematic chain and restoring the mechanical equilibrium of the lower extremities. We plan to conduct a randomized controlled trial (RCT) to evaluate the clinical efficacy of Tuina therapy based on hip-knee-ankle synergy in the kinematic chain of lower extremities in patients with KOA and explore the potential effect of synergistic therapy.

## Methods

### Study Design

**Figure 1:**
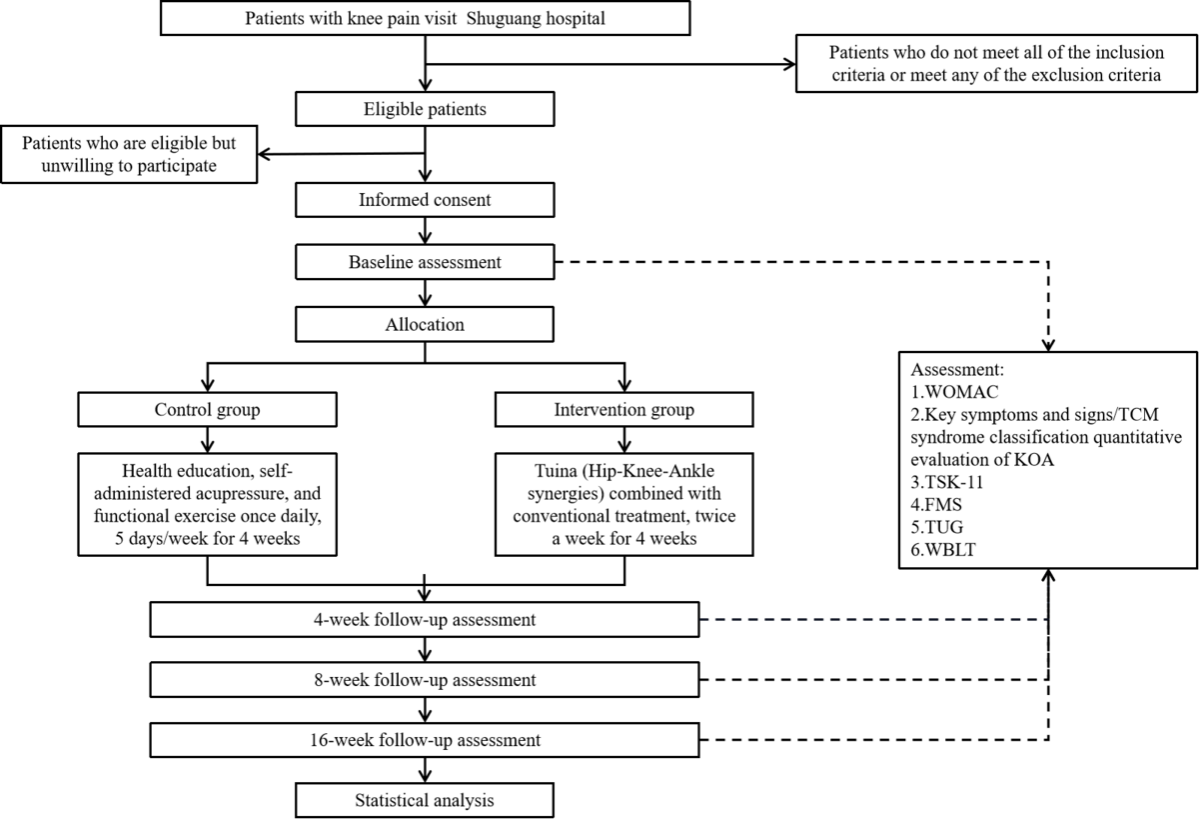
Flowchart of the study. WOMAC, Western Ontario and McMaster Universities Arthritis Index; TSK-11, Tampa Scale of Kinesiophobia; FMS,: Functional Movement Screen; TSK-11: 11-item Tampa Scale of Kinesiophobia; TUG,: Timed Up and Go; WBLT,: Weight Bearing Lunge Test; WOMAC: Western Ontario and McMaster Universities Osteoarthritis Index.

A single-center, single-blind RCT has been registered with the International Traditional Medicine Clinical Trial Registry (ITMCTR2024000293). The study center is Shuguang Hospital affiliated with the Shanghai University of Traditional Chinese Medicine, a large tertiary hospital with 2 distinct campuses located in different regions of Shanghai, each serving diverse patient populations. The flowchart in Figure 1 illustrates the process of the study.

### Participants

All participants will be recruited from among patients with KOA visiting Shuguang Hospital affiliated with the Shanghai University of Traditional Chinese Medicine. Patients will first be enrolled based on their basic information, medical history, X-ray results, and WOMAC (Western Ontario and McMaster Universities Osteoarthritis Index) scores. Patients who are willing to participate in this trial and demonstrate an understanding of the objectives and methods of the study by providing signed informed consent will then be randomly assigned to 2 groups: a conventional treatment (control) group and a Tuina+conventional treatment (intervention) group.

At baseline, some information will be collected in order to account for potential confounding variables, including basic information (name, sex, age, height, weight, telephone number, profession). We will calculate the patients’ BMI using their height and weight. Physical activity levels will be assessed and recorded using a 3-tier classification (light, moderate, and vigorous). Additionally, we will document the types of comorbidities and medication use, including type, dosage, and frequency. These variables will be included in multivariate analyses to adjust for their potential confounding effects on pain perception and functional outcomes.

All participants will be evaluated 4 times: at baseline and at 4-, 8-, and 16-week follow-up.

The inclusion criteria are as follows:

- A diagnosis of KOA according to American College of Rheumatology criteria [17]
- Average pain score in WOMAC≥4
- An abnormal lower limb line on the lower limb length X-ray
- The patient’s age is 20-65 years, male or female.
- No other treatment in the past 4 weeks that may interfere with the results of this study

In addition, patients with unilateral KOA will be included in the study.

The exclusion criteria are as follows:

- Patients with severe bone and joint diseases, such as rheumatoid arthritis, osteoporosis, gout, or an active knee infection in the past 12 months
- Patients with cardiovascular, cerebrovascular, liver, kidney, hematopoietic, digestive, or mental illness or other conditions besides KOA that cause more restriction or pain in activities such as sitting, standing, or walking
- Pregnant and lactating women who cannot complete the radiological examination
- Patients who cannot understand and complete research related to various scales and provide informed consent
- Other conditions that the investigator considers inappropriate to participate in the study

In addition, participants will be removed from the study under the following conditions:

- Patients with severe adverse reactions or complications
- Patients who do not receive the treatment as initially intended or are prescribed therapy elsewhere without the investigator’s authorization

### Sample Size Calculation

Sample size calculation was based on WOMAC scores (the primary outcome). The sample size formula for comparing means between 2 groups in an RCT was used to calculate the sample size. With test levels set at α=.05 (2-sided) and β=0.1, according to the results of a high-quality RCT [18], the SD of the WOMAC score was 44.7, and the mean difference between the 2 groups after the intervention was 32.6, which was replaced by the sample size calculation formula, n=39.47. During the study, unforeseeable situations may emerge, which could lead to the loss of some data. In anticipation of this, the dropout rate has been set to 20% [19,20], and with n=48, we will include 36 participants in each group (total 96 participants).

### Randomization, Allocation, and Blinding

Randomization will be performed using IBM SPSS Statistics 27.0 and manual assistance. Prior to the study, a randomization specialist will be assigned to generate a random set of numbers based on numbers 1-96 using IBM SPSS Statistics 27.0. After the numbers are sorted from small to large, the 96 cases will be randomly divided into the intervention group and the control group, and the numbers and corresponding groups will be sent to the researchers. Recruited patients who meet the inclusion criteria will be listed in a table and numbered in order of enrollment. The current caseload will be determined according to the grouping generated by randomly assigned personnel. The lists of the 2 groups will be sorted into tables and sent to the actual operators, who will be informed of the specific methods of operation of each group. This method can reduce unnecessary communication between researchers and randomization specialists in the grouping process as far as possible in order to reduce potential bias from researchers’ subjective intentions in participant grouping.

We have established rigorous assessment protocols and guidelines. An independent assessor, blinded to participants’ group allocations and unaware of the intervention methods used for the 2 groups, will conduct all outcome evaluations. Upon completion of the study, the assessor will be requested to estimate participants’ group assignments. Subsequently, their estimations will be compared with the actual group allocations. This procedure aims to evaluate the effectiveness of the blinding process. Should any breach of blinding be identified, we will thoroughly account for it and provide a comprehensive explanation within the data analysis and interpretation of results.

Information about the existence of another intervention group will not be disclosed to the participants, and they will be strictly instructed not to disclose any information related to their intervention to the assessor.

### Intervention

We will explain the details of our study to the participants before they join our study.

#### Control Group (Conventional Treatment)

According to the “Basic treatment” part of the *Guidelines for TCM Diagnosis and Treatment of Knee Osteoarthritis* (2020 edition) [21], we will include health education, weight management, functional exercise, and self-administered acupressure [22]. We will distribute health education manuals, provide online and offline guidance for patients, popularize KOA-related health knowledge to patients, and help them understand the importance of daily KOA protective measures and precautions through popular science health education. In this group, patients will be taught self-administered acupressure and functional exercise once daily, 5 days/week for 4 weeks.

#### Self-Administered Acupressure

First, patients will form a claw-like shape with 1 hand and use it to squeeze the quadriceps muscle along the front of the thigh, moving along the pathways of the liver, spleen, stomach, and gallbladder meridians.

Second, they will use their fingers/thenar/hypothenar to press the acupoints (ST34, ST35, ST36, SP9, SP10, GB34, EX-LE2, and EX-LE4) briskly and then release with moderate pressure repetitively for about 1 minute per acupoint.

Third, they will rub their hands together until they become warm. Subsequently, they will place their hands around the knee joints and perform gentle, repeated kneading motions until the knee joints experience a sensation of slight warmth.

#### Functional Exercise

Participants will perform straight leg-raising exercises, 3-5 sets per day, with 10-15 repetitions per set. They will also perform wall squats, 3-5 sets per day, commencing with 30-60 seconds per squat and progressing to 90-120 seconds as muscle strength increases, with the knee angle maintained between 120°and 150°. Furthermore, they will perform knee flexion and extension resistance exercises, 3-5 sets per day, with 10-15 repetitions per set.

#### Intervention Group (Tuina Plus Conventional Treatment)

**Figure 2:**
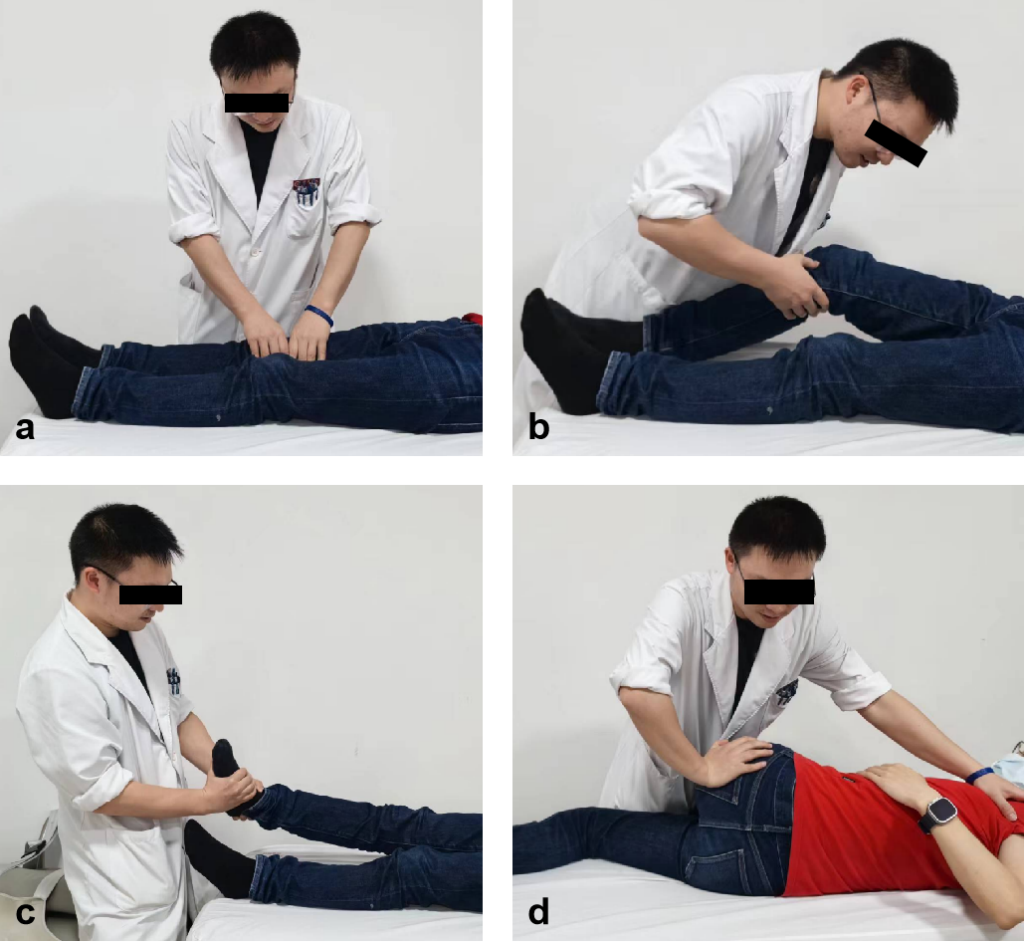
Tuina therapy with hip-knee-ankle synergies: (a) Relax the muscles of the lower extremity (with emphasis on the region surrounding the knee), (b) perform arthrokinetic mobilization and adjustment of the knee, (c) perform arthrokinetic mobilization and adjustment of the ankle, and (d) perform arthrokinetic mobilization and adjustment of the hip.

In this group, the treatment regimen will also receive Tuina therapy based on hip-knee-ankle synergy (Figure 2). The frequency of treatment will be twice a week for 4 weeks. We have established a scientific, normative, standardized work process, operating procedures, and quality standards. All treatment will be performed by registered Tuina doctors, who have clinician qualifications and have been engaged in Tuina clinical work for more than 5 years. All of have graduated with Tuina as their major and have been trained and assessed with Tuina's standard operating procedures (SOP) for this study.

First, the patient will lie in a supine position, with legs straight and relaxed. The doctor will relax the soft tissues of the groin, thigh, knees, and lower legs in turn, including the quadriceps femoris muscle, the tibialis anterior muscle, and soft tissues around the patella. The degree of manual relaxation will be determined based on the patient’s sensation of slight soreness and distension.

Second, the patient will keep lying in a supine position. Acupressure will be administered to the points around the knee. The patella will be mobilized, and the cordage, patellar ligament, medial and lateral retinaculum, and medial and lateral collateral ligament will be relaxed. Depending on the actual situation, knee adjustment manipulation techniques in a seated or a supine position will be performed, when necessary. Next, the doctor will rub the knee and transfer heat.

Third, the patient will keep lying in a supine position. The doctor will hold the patient’s ankle with both hands and place the thumb on the lateral or medial malleolus suture and press, while the other 4 fingers will be fixed, rotating and shaking the foot several times. The ankle will be pulled outward and downward with both thumbs, and the ankle joint will be held and the lower limb stretched.

Finally, the patient will lie in a prone position, the doctor will relax the muscles of the buttocks, thighs, and calves in turn, including the gluteus maximus, gluteus medius, piriformis, biceps femoris, and gastrocnemius muscles. The handling will not be too heavy. The doctor will measure the degree of swelling and numbness. Depending on the actual situation, hip joint adjustment manipulation techniques will be performed, when necessary.

### Outcome Measures

To enhance patient compliance and mitigate potential bias or data loss from frequent in-person visits, we have opted to evaluate WOMAC, Key Symptoms and Signs/TCM Syndrome Classification Quantitative Evaluation of KOA [21], and TSK-11 [23] scores at 4 time points (baseline and 4-, 8-, and 16-week follow-up), while assessing Functional Movement Screen (FMS) [24,25], Timed Up and Go (TUG) test [26,27], and Weight Bearing Lunge Test (WBLT) scores at 2 time points (baseline and 4-week follow-up).

#### Primary Outcome

##### Western Ontario and McMaster Universities Osteoarthritis Index

WOMAC [18,28] is a commonly used outcome measure for OA, and it will be applied at baseline and at 4-, 8-, and 16-week follow-up. WOMAC is composed of 24 items in 3 subscales (5 for pain, 2 for stiffness, and 17 for physical function). Each item is rated on a scale of 0-10 (with higher scores indicating worse pain, function, and stiffness), and total scores range from 0 to 240. WOMAC scores are classified as mild (<80), moderate (80-120), and severe (>120). Assessing the changes in WOMAC scores before and after the intervention will enable us to evaluate the specific effects of Tuina on pain relief and functional improvement in patients with KOA.

#### Secondary Outcomes

##### Key Symptoms and Signs/TCM Syndrome Classification Quantitative Evaluation of KOA

This evaluation focuses on assessing the degree of pain, swelling, and accompanying symptoms in patients with KOA. It will be used to evaluate the short- or midterm clinical efficacy of Tuina in KOA and will be applied at baseline and 4-, 8-, and 16-week follow-up. It. This will allow us to observe the effects of Tuina on KOA from a TCM perspective, offering a comprehensive understanding of its therapeutic effects.

##### Tampa Scale of Kinesiophobia

This tool is an 11-item questionnaire based on a 7-point Likert scale (1=strongly disagree, 2=disagree, 3=agree, 4=strongly agree) that is used to evaluate fear of exercise injury in patients with chronic pain and will help us assess whether Tuina can reduce patients’ fear of movement and enhance their rehabilitation participation. It will also be applied at baseline and at 4-, 8-, and 16-week follow-up. Higher TSK-11 scores indicate a greater fear of pain, and a score above 24 is considered high-level kinesiophobia.

##### Functional Movement Screen

**Figure 3:**
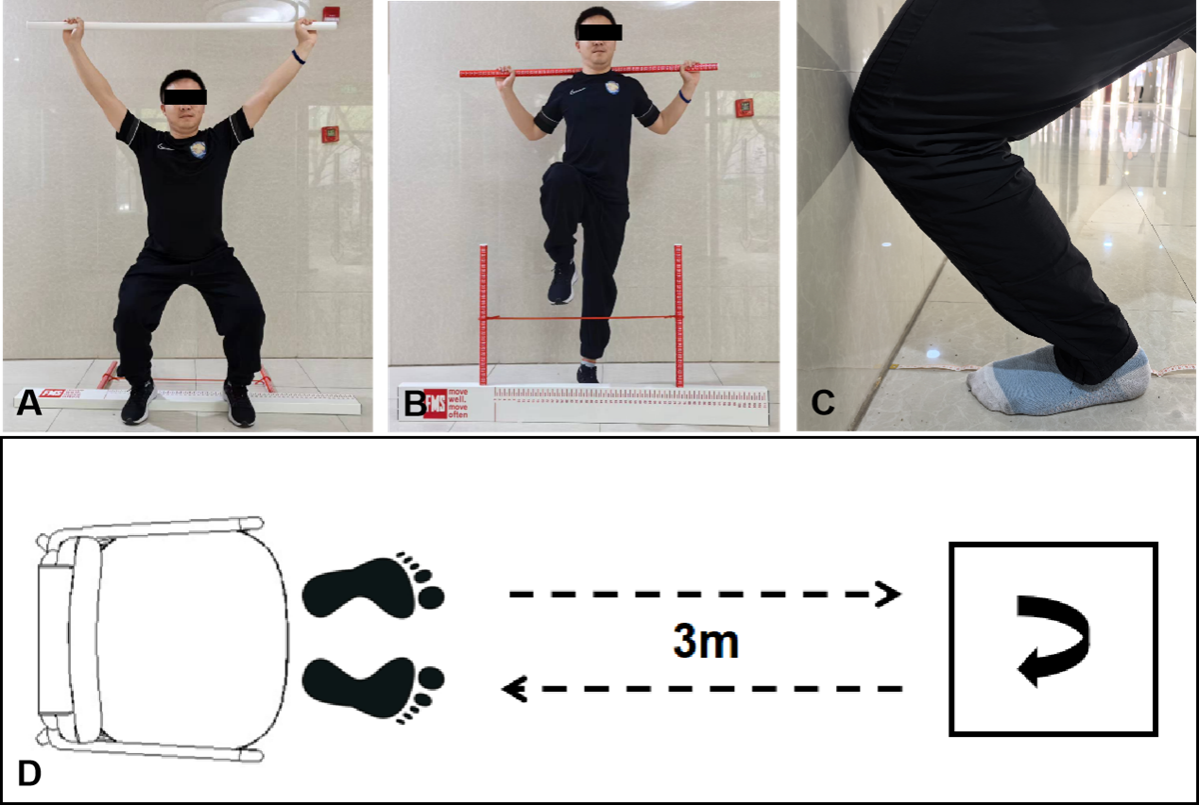
Action demonstration: (A, B) FMS, (C) WBLT, and (D) TUG test. FMS: Functional Movement Screen; TUG: Timed Up and Go; WBLT: Weight Bearing Lunge Test.

The FMS Figure 3A and B is a standardized field-expedient test cell designed to assess the quality of movement and has been used clinically in prescreening and in sports injury research. It will be used to assess objective physical function at baseline and 4-week follow-up. This tool uses 7 functional movements to assess movement pattern deficits and asymmetries, including deep squat, hurdle step, inline lunge, shoulder mobility, active straight leg raises, trunk stability pushup, and rotary stability. By assessing overall movement patterns, the FMS can comprehensively evaluate the impact of Tuina on patients’ motor function recovery. In this study, we will only evaluate the deep squat and hurdle step.

##### Timed Up and Go

The TUG Figure 3D test is a simple and effective way to evaluate a patient’s ability to rise from a chair, walk, and turn, which is clinically applicable and reliable across multiple populations. Given that patients with KOA often experience walking difficulties and balance impairments, the TUG test offers a direct measure of functional mobility highly relevant to daily activities. By assessing TUG scores before and after our intervention, we can assess Tuina’s effects on mobility and balance, thereby verifying its clinical effectiveness. It will be used to assess objective physical function at baseline and 4-week follow-up.

At the beginning of the test, the patient will sit in a chair with their feet flat on the ground and their hands on their knees. We will ask them to get up, walk 3 m, turn around, and return to the chair. During this procedure, the evaluator will record the time it takes for the patient to complete the test. The longer the time, the higher the risk.

##### Weight Bearing Lunge Test

The WBLT Figure 3C is an ankle dorsiflexion activity under closed kinetic chain exercise of the knee joint, and its reliability and effectiveness have been widely recognized. It will also be applied at baseline and at 4-, 8-, and 16-week follow-up. Researchers have identified a potential relationship between the limited range of dorsiflexion motion in the ankle and knee kinematics, which may increase the risk of knee injury [29].

Before the test, a tape measure with a scale mark will be placed on the floor. During the test, the patient will be asked to face the wall, stand with one foot in front of the other, step on the tape on one side, and point the toes vertically toward the wall (initially at an appropriate distance from the wall), keeping the longitudinal axis of the foot in the center of the tape. They will keep the heel close to the ground and gradually move the knee forward. If the knee touches the wall, they will slowly move the knee and then the leg back and then repeat bending the knee toward the wall until the heel is about to leave the ground.

The assessor will measure the distance between the toes and the wall 3 times, calculate the average of the measurements, and record the results.

### Cointervention Assessments

Patients will be advised not to receive other KOA interventions (eg, acupuncture, intraarticular injection) during the study. Additional interventions will be meticulously recorded.

### Safety Assessments

We will also pay close attention to adverse events. Throughout the trial period, any adverse events or phenomena observed will be carefully recorded in the designated case report form (CRF). This documentation will include the precise timing of the event, the accompanying symptoms, the severity of the condition, its duration, the therapeutic measures implemented, and the outcome. The research team will carefully analyze the correlation between adverse events and the required intervention, recording their details, signs, and precise dates for future reference.

### Statistical Analysis

IBM SPSS Statistics 27.0 will be used for all data analyses. The different baseline characteristics of the 2 groups will be described. Data will be presented as the mean (SD) or the median (IQR) for continuous variables and as frequency distributions for categorical variables. The 2-sample *t* test for quantitative data or the chi-square test for qualitative data will be performed as a homogeneity test, and covariance analysis will be performed if an adjustment is needed for a baseline characteristic. Analysis will focus on whether statistically better treatment outcomes can be achieved in the intervention group. *P*<.05 is considered statistically significant and the tests are 2-sided. If data loss occurs, we will use multiple imputations to create several datasets to replace missing values and reduce bias. For repeated-measures data across 4 time points, we will apply linear mixed effects models to handle unequal intervals and account for individual random effects. In addition, repeated-measures ANOVA will test the statistical significance of variable differences between time points. Combining these methods will help reveal variable trends over time and provide reliable statistical support for the findings.

### Ethical Considerations

This protocol was approved in March 2025 by the Medical Ethics Committee of Shuguang Hospital affiliated with the Shanghai University of Traditional Chinese Medicine (approval number 2024-1560-143-01). All participants will provide signed informed consent, and upon completion of the study, they will receive a subsidy of 100 yuan (~US $14). Patient consent for publication is not applicable.

Data management is extremely important. In this study, electronic data will be stored on a dedicated computer in our research center. This computer will be protected by a password, a firewall, and an intrusion detection system, which can effectively prevent unauthorized access and data breaches, ensuring data security. In addition, data will be regularly backed up according to a set schedule to prevent loss. They will also be stored using a unified file-naming convention and directory structure for easy retrieval and management. Paper-based data, including CRFs, will be stored in locked filing cabinets at our research center. They will be organized and clearly labeled, and modifications will be recorded with the original content retained. Only authorized personnel will have access. Any personal information will be anonymized. Data will be retained as required by regulations to ensure integrity, traceability, and confidentiality.

Data access will be strictly controlled based on research roles and responsibilities. Principal researchers will have unrestricted access to perform their duties effectively. Other research team members will be granted access solely to the data essential for their specific tasks. Additionally, the Ethics Committee and regulatory authorities will have the right to review the data in accordance with relevant laws and ethical guidelines to ensure compliance with research standards. Additionally, all data access activities will be logged and subject to periodic review.

After the study, data will be properly stored following institutional and regulatory requirements. After the retention period, all data and records will be uniformly destroyed to ensure the long-term maintenance of participant privacy.

### Quality Assurance

The project manager will be exclusively responsible for conducting all quality assurance. Before the study, all researchers are expected to undertake a comprehensive and uniform protocol-specific training program, encompassing the study protocol, CRF filling, data collection, data entry, and standard of functional tests. During the study, the project manager will conduct periodic inspections on all study components to ensure rigorous adherence to the study protocol and accurate documentation of the research data. Functional tests will be evaluated by specific researchers. The protocol will remain unaltered during the study period.

## Results

We have successfully recruited 55 participants for the study, including those undergoing the intervention, those in follow-up, and those who have completed the study, with recruitment expected to be completed by September 2025. Data analysis has not yet been initiated. Upon completion of the study, the results will be submitted to a peer-reviewed journal for rigorous evaluation.

## Discussion

### Summary

The knee joint serves as a significant component of lower limb movements and acts as a pivotal link in the lower limb kinematic chain [30]. In the framework of kinematic chain theory [31,32], the lower limb is considered a harmonious and integrated unit, wherein the cooperative interplay of hip, knee, and ankle articulations is postulated as the vital factor for ensuring postural equilibrium and optimal gait function in the human body. The 3 joints are closely related, and any aberration in any of the links may culminate in the derangement of the lower limb’s force line and the diminution of joint stability, potentially leading to irregular muscle contraction patterns, abnormal joint stress, and irregular neuromuscular interaction. This condition eventually leads to incongruous patterns of lower limb movement. Therefore, in the treatment of KOA, a holistic approach should be implemented, focusing on the integrity and functionality of the entire musculoskeletal system rather than relying excessively on local interventions.

Holism is a fundamental characteristic of TCM. The application of holistic concepts and differentiation of syndromes in diagnostic and therapeutic processes is a central concern in clinical practice. The inclusion of Tuina therapy in KOA management represents a frequently used and effective treatment strategy. Moreover, Tuina can effectively complement the treatment regimen for the hip, knee, and ankle joints. Therefore, we will develop a comprehensive clinical trial to evaluate the efficacy of Tuina therapy based on hip-knee-ankle synergy in the treatment of KOA.

### Strengths and Limitations

To the best of our knowledge, this study is the first RCT to explore the clinical efficacy of Tuina therapy based on hip-knee-ankle synergy in patients with KOA. Our study will adopt a comprehensive approach to functional assessment, integrating both objective measurements and subjective patient-reported outcomes. This dual methodology will allow us to not only quantify improvements through standardized evaluation protocols but also capture patients’ personal experiences and perceptions of their recovery. By combining these perspectives, we seek to offer a more nuanced and complete assessment of how intervention affects physical function. The primary outcome measure used in this study is WOMAC, a validated and reliable tool specifically designed to assess the presence and severity of lower extremity pain, stiffness, and functional limitations associated with OA [33]. Numerous previous studies have used WOMAC in behavioral intervention trials for osteoarthritis, validating its use as an appropriate indicator of clinical efficacy in this context. Key Symptoms and Signs/TCM Syndrome Classification Quantitative Evaluation of KOA can assess the degree of pain, swelling, and accompanying symptoms. TSK-11 can accurately predict the degree of physical disability in this patient population [34]. The TUG test represents abilities related to ambulatory transitions, leg strength, and balance [35], the FMS is crucial in assessing the quality of movement, and the WBLT is used to assess ankle dorsiflexion activity.

We anticipate that the results of this study will demonstrate the effectiveness of Tuina therapy based on hip-knee-ankle synergy in improving pain relief, functional outcomes, and overall quality of life for patients with KOA. For example, we expect to observe statistically significant improvements in WOMAC scores, indicating reduced pain, stiffness, and functional limitations. We also expect to observe statistically significant improvements in TSK-11 scores, indicating reduced fear of movement in patients. Additionally, we expect to observe statistically significant improvements in TUG, FMS, and WBLT outcomes. Such improvements would suggest potential enhancements in patients’ physical activity and balance abilities. These potential findings would provide strong evidence supporting the integration of Tuina therapy into conventional treatment approaches for KOA, emphasizing the importance of considering the entire musculoskeletal system rather than focusing solely on localized treatments.

Although the study design inherently creates a potential imbalance in treatment dosage between the 2 groups, which may lead to differences in compliance and potentially affect the comparability of the results, several measures have been implemented to minimize the bias introduced by this imbalance. The assessor will be blinded to participants’ group allocations and remain unaware of the intervention methods used for the 2 groups. Additionally, information about the existence of another intervention group will not be disclosed to the patients, and they will be strictly instructed not to disclose any information related to their intervention to the assessor.

Our study was conducted in a single medical institution, a large tertiary hospital with 2 campuses in different regions of Shanghai that serve diverse patient populations. Although this setup partially mitigates the limitations of a single-center study, it does not fully replicate the diversity of multisite studies conducted across varied geographical areas. Future research should investigate the intervention’s effectiveness in multiple health care settings and populations to better understand its generalizability and real-world applicability.

### Conclusion

In summary, we believe this study will offer valuable insights into the clinical efficacy of combining Tuina therapy based on hip-knee-ankle synergy for patients with KOA. If our anticipated results are realized, they could significantly influence the development of more comprehensive and effective treatment protocols in clinical practice. Moreover, the findings may enhance our understanding of KOA’s underlying mechanisms and the benefits of holistic treatment approaches.

## Abbreviations

CRF: case report form
FMS: Functional Movement Screen
KOA: knee osteoarthritis
OA: osteoarthritis
RCT: randomized controlled trial
TCM: traditional Chinese medicine
TSK-11: 11-item Tampa Scale of Kinesiophobia
TUG: Timed Up and Go
WBLT: Weight Bearing Lunge Test
WOMAC: Western Ontario and McMaster Universities Osteoarthritis Index
